# Maternal Diabetes and Fetal Programming Toward Neurological Diseases: Beyond Neural Tube Defects

**DOI:** 10.3389/fendo.2018.00664

**Published:** 2018-11-13

**Authors:** Berenice Márquez-Valadez, Rocío Valle-Bautista, Guadalupe García-López, Néstor Fabián Díaz, Anayansi Molina-Hernández

**Affiliations:** ^1^Department of Physiology and Cell Development, Instituto Nacional de Perinatología Isidro Espinosa de los Reyes, Mexico City, Mexico; ^2^Department of Physiology, Biophysics and Neurosciences, Centro de Investigación y de Estudios Avanzados del Instituto Politécnico Nacional, Mexico City, Mexico

**Keywords:** diabetes, pregnancy, fetal programming, neurological disorders, psychiatric disease

## Abstract

The purpose of this review was to search for experimental or clinical evidence on the effect of hyperglycemia in fetal programming to neurological diseases, excluding evident neural tube defects. The lack of timely diagnosis and the inadequate control of diabetes during pregnancy have been related with postnatal obesity, low intellectual and verbal coefficients, language and motor deficits, attention deficit with hyperactivity, problems in psychosocial development, and an increased predisposition to autism and schizophrenia. It has been proposed that several childhood or adulthood diseases have their origin during fetal development through a phenomenon called fetal programming. However, not all the relationships between the outcomes mentioned above and diabetes during gestation are clear, well-studied, or have been related to fetal programming. To understand this relationship, it is imperative to understand how developmental processes take place in health, in order to understand how the functional cytoarchitecture of the central nervous system takes place; to identify changes prompted by hyperglycemia, and to correlate them with the above postnatal impaired functions. Although changes in the establishment of patterns during central nervous system fetal development are related to a wide variety of neurological pathologies, the mechanism by which several maternal conditions promote fetal alterations that contribute to impaired neural development with postnatal consequences are not clear. Animal models have been extremely useful in studying the effect of maternal pathologies on embryo and fetal development, since obtaining central nervous system tissue in humans with normal appearance during fetal development is an important limitation. This review explores the state of the art on this topic, to help establish the way forward in the study of fetal programming under hyperglycemia and its impact on neurological and psychiatric disorders.

## Introduction

Diabetes is a group of metabolic diseases characterized by deficient insulin secretion and/or action which leads to hyperglycemia, and, in turn, to abnormal metabolism of carbohydrates, fats, and proteins in insulin target tissues (1). Depending on pathogenesis, diabetes can be classified as type 1, type 2, gestational diabetes mellitus (GDM), or other types of diabetes. Worldwide, ~5–10% of diabetic patients have type 1 diabetes, in which complete insulin deficiency stems from β-cell autoimmune destruction as a result of genetic susceptibility or viral antigens. Type 2 affects 90–95% of diabetic patients and is characterized by either insulin resistance or defective secretion. Other, less common, types of diabetes are associated with monogenetic defects in β-cell function, pancreatic injuries, and drug abuse, among other causes (1).

GDM is commonly diagnosed in the second or third trimester of pregnancy, and its prevalence varies between 1 and 17% depending on the studied population and the diagnostic test used (1, 2). Due to the ongoing epidemic of obesity and diabetes in women of childbearing age, the American Diabetes Association (ADA) has established that women with GDM risk factors must be tested for diabetes during the second trimester of pregnancy, using standard diagnostic criteria. Furthermore, that diabetes in women in the first trimester should be classified as type 2 diabetes (1). However, pregnant women with no risk factors can develop diabetes as early as the first trimester (3–5), making it evident that earlier testing is required in some cases.

**Figure 1:**
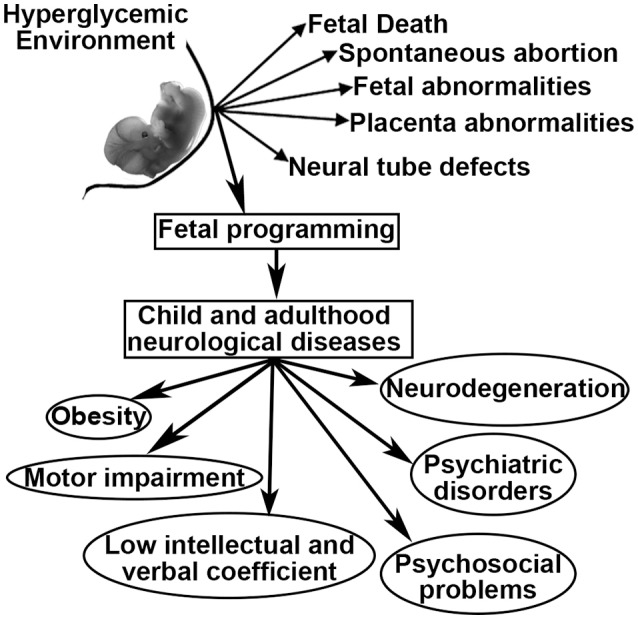
Possible outcomes related to intrauterine hyperglycemia.

As a result of GDM, women and their offspring confront a series of problems including fetal death, spontaneous abortion, congenital malformations, fetal-placental abnormalities, and altered fetal programming (6–8). Fetal programming is defined as the development of pathologies during childhood and adulthood that originate during fetal development (9). In this sense, maternal diabetes has been linked to offspring that develop obesity, diabetes, neurodegenerative and psychiatric diseases, as well as low intellectual and verbal coefficients, language and motor impairments, attention deficit with hyperactivity disorder, and problems in psychosocial development (10–15). However, many of these relationships remain unclear, have not been well-studied and have not been related to fetal programming (Figure 1).

During this review, we realized that existing information regarding the mechanisms of action between hyperglycemia during fetal development and the outcomes is limited. Most reports are focused on *in utero* hyperglycemia and neural tube defects (NTD). Also, studies on viable “normal” offspring and strategies to prevent the effects of maternal diabetes are scarce; an understandable problem given that several extrinsic and intrinsic factors (including embryo susceptibility, among others), may contribute to CNS fetal programming in specific cell types, locations, and times.

### Bibliographic search

We searched for experimental and clinical evidence regarding the effect of hyperglycemia on the development of the CNS and fetal programming related to neurological diseases, but excluding evident NTD. We searched PubMed (https://www.ncbi.nlm.nih.gov/pubmed) for studies on humans or other animals, published in English in a variety of article types (classical article, clinical study, comparative study, evaluation studies, journal article, meta-analysis, and technical report) published between 1990/01/01 and 2018/01/31. We used a combination of the following keywords: maternal diabetes or pregnancy+hyperglycemia, in combination with neurological fetal programming, neurological outcomes, fetal neural development, or neural tube.

From a total of 525 articles, 76 remained after we eliminated those that contained the phrase “neural tube defect,” those that were not related to CNS development and/or function, duplicated articles, and those published in a language other than English.

## Obesity and diabetes

Childhood obesity and type 2 diabetes are closely related to GDM. A systematic review that included 20 observational studies involving a total of 26,509 children showed that maternal hyperglycemia (GDM and type 1 diabetes) was associated with obesity and abnormal glucose tolerance in offspring. Interestingly, while higher body mass index was reported for the children of GDM mothers during childhood, the same was reported from prepuberty to adolescence in children from mothers with type 1 diabetes. Furthermore, offspring from GDM mothers had high 2-h plasma glucose from prepuberty to early adulthood, and those from mothers with type 1 diabetes had a high rate of type 2 diabetes from years 2 to 5 and early adulthood (16). On the other hand, the effect of diabetes during gestation in offspring can be generationally transmissive through the maternal line. Hanafi et al. (17) showed that rats with grand-maternal diabetes showed impaired glucose sensing, increased oxidative stress, insulin resistance, and impaired glucose tolerance in F1 and F2, with more prominent effects in F2.

In an effort to study the mechanisms involved in the development of obesity in offspring from diabetic mothers, the plasma content of hormones involved in food intake and energy expenditure were measured in an Austrian cohort of children with a mean age of 6 years (male:female = 36:40) born from mothers with GDM, pre-gestational diabetes, and nondiabetic women. No differences were found in the plasma content of hormones involved in food intake and energy expenditure such as ghrelin, leptin, adiponectin, neuropeptide Y (NPY), peptide YY, and growth differentiation factor 15. However, using multiple regression analysis, the authors found that body mass index, leptin, and GDF-15 had independent effects on insulin resistance (18). It is worth mentioning that both GDF-15 and leptin are synthesized by white adipose tissue; the first decreases food intake, while the latter suppresses appetite and increases energy expenditure (19, 20). Furthermore, leptin is a hormone that acts via receptors in the hypothalamus, and the activation of leptin receptors in pro-opiomelanocortin (POMC) and agouti-related peptide (AgRP)/NPY neurons within the arcuate nuclei (ARC) lead to increased AgRP and POMC expression reducing food intake (19, 21). The absence of changes in plasma leptin in obese children from GDM, but its relation with body mass index may be explained through an impaired function in the leptin receptor expressed in the CNS, a phenomenon called, leptin resistance.

POMC is the precursor of α-melanocortin stimulating hormone, which in turn (through the activation of type 3 and 4 melanocortin receptors) is the most important component of the network responsible for controlling appetite, energy expenditure, glucose homeostasis and lipid metabolism in the hypothalamus (19, 22, 23). This peptide functions as an anorexigenic factor in ARC and paraventricular nuclei (PVN), predominantly reducing appetite (24). AgRP and NPY are orexigenic factors inducing hyperphagia and obesity (25–27).

The interaction between peripheric leptin and the melanocortin systems in the ARC and PVN is essential for the circuits that regulate food intake, energy expenditure, glucose, and lipid metabolism. Furthermore, it can be suggested that changes in hypothalamic fetal ontogenesis could be taking place in the diencephalon exposed to hyperglycemia, affecting the postnatal function of hypothalamic circuits because of the effects reported at early and late development in offspring from GDM and Type 2 diabetes mothers on leptin level, glucose homeostasis and BMI, mentioned above (18, 16). Indeed, several studies have shown that high glucose levels promote an inadequate organization of hypothalamic ARC and PVN, as well as malformation of the ventromedial hypothalamic nucleus in rats (28–31). Moreover, chicken embryos exposed to high glucose concentrations (30 mM) have lower glucose tolerance in neurons located in the hypothalamic infundibulum the equivalent anatomic area of the ARC (32).

**Figure 2:**
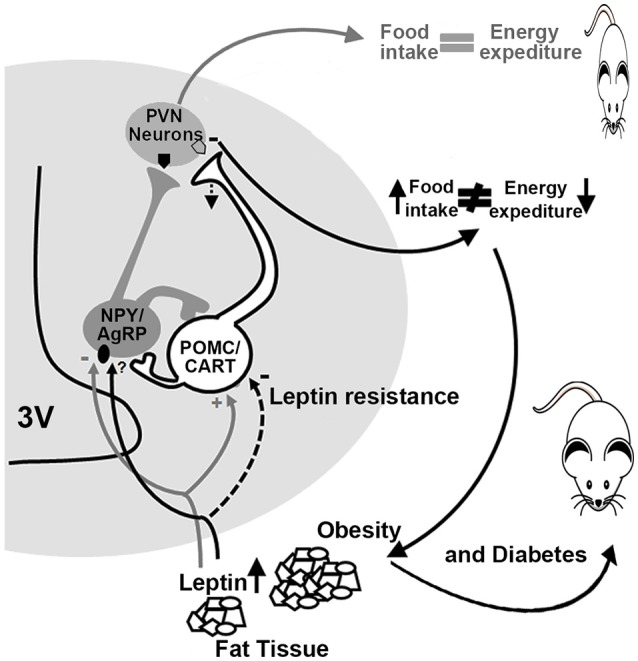
Altered energy and food intake balance in diabetic dams offspring. In normal conditions (gray lines and arrows), leptin activates POMC/CART neurons (pro-opiomelanocortin/cocaine- and amphetamine-regulated transcript) and inhibits NPY/AgRP neurons (neuropeptide Y/agouti-related protein) promoting satiety and a balance between food intake and energy expenditure. In diabetic offspring (black lines and arrows), a decrease in the activation of POMC/CART neurons takes place due to leptin resistance, which reduces melanocortin release at the PVN (paraventricular nuclei), promoting an increase in food intake leading to obesity and diabetes. 3V, third ventricle.

On the other hand, the expression of hypothalamic neuropeptides in sheep ARC exposed to intrauterine hyperglycemia increases the expression of POMC and the cocaine and amphetamine-related transcripts (CART) at 81 and 140 days of gestation (33, 34). Such changes may affect energy balance regulation in later life, affecting food intake and energy balance. To our knowledge, there is no postnatal evidence of an altered pathway for central energy balance in sheep, but there are some clues provided in the murine model. Offspring from diabetic rats and mice have increased susceptibility to body weight dysregulation and obesity due to: increased expression of the orexigenic (NPY and AgRP) and decreased expression of the anorexigenic (α-MSH) peptides in the ARC (35, 30), leptin resistance in 10-day old pups, decreased fiber density of AgRP and α-MSH peptides, as well as in the PVN, and increased food intake and body weight (31). These findings suggest that offspring born from diabetic dams showed leptin resistance in first-order neurons within the ARC, less synaptic transmission into the PVN and, consequently, obesity (Figure 2).

Altered levels of morphogens and transcription factors important in hypothalamic organization may affect hypothalamic fetal development under hyperglycemic conditions. During early fetal development, the hypothalamus emerges from the diencephalon, and its adequate formation depends on the precise regulation of molecular and cellular mechanisms orchestrated by regional morphogenetic organizers in the neural tube. The diencephalon is separated from surrounding regions by the influence of organizing signals (morphogens) such as Wingless/integrins, Sonic hedgehog (Shh), bone morphogenetic proteins and fibroblast growth factors, and later by the expression of the thyroid transcription factor 1 (Nk × 2.1, a downstream gene of Shh) and a key factor for hypothalamic neuron development (36, 37).

Interestingly, Shh has been evaluated by *in situ* hybridization and qRT-PCR during brain development in embryos from streptozotocin-induced diabetic mice, at embryo day (E) 8.5 and E11.5. Normally, at E8.5 Shh expression is restricted to the ventral medial plate in the forebrain, but its domain is expanded and its expression increases in embryos from diabetic mice. At E11.5 in control animals, Shh expression is localized in the ventral diencephalon and telencephalon, but in the diencephalon from diabetic animals its expression is stronger and expanded toward the dorsal telencephalon. Thus, there is an increased and expanded expression of Nk × 2.1 to the telencephalon (38). In addition, an increase in cell proliferation and neurogenesis has been reported in ventral telencephalon/diencephalon in E11.5 to diabetic mice (39).

These data suggest that early changes in the ventral-dorsal patterning and increased neurogenesis are contributing to defects in the fetal and postnatal hypothalamus in murine, chicken and sheep models; an aspect worthy of further study.

## Cognition

Cognition is involved in the regulation of emotional and social cues, including the formal measures of intelligence such as memory and attention. Cognitive functions studied in infants from diabetic mothers and offspring from different animal models include language (animal communication), learning, memory, motor coordination, perception, and problem-solving. All of these are functions that are coordinated in a complex manner by different anatomical structures such as the cerebral cortex, amygdala, hippocampus, and basal ganglia. However, the relationship between diabetes during pregnancy and impaired cognitive function after birth remains controversial. Some studies in humans have shown that maternal diabetes contributed to cognitive dysfunction in school-age children, which has been associated with changes in cell migration and differentiation during brain development (12, 40, 41). For example, Ornoy et al. (12) reported that children under 9 years old born to GDM women had lower scores in verbal tasks and fine and gross motor skills. Bolaños et al. (42) found that *in utero* hyperglycemia was associated with a lower average IQ and poor performance in working memory skills, such as graphic and visuospatial tasks, in children from 7 to 9-years-old born to control and GDM women: They concluded that GDM leads to minor neurological deficits in children (42).

Working memory is the result of proper coordination between the prefrontal cortex and hippocampus; structures that arise from the dorsal telencephalon during embryo development.

Hyperglycemia during hippocampal development decreases synaptic plasticity and reduces memory durability in male rats (43, 44), presumably due to a delay in normal hippocampal development regulated by insulin and insulin growth factor-1 receptors that lead to structural, behavioral, and cognitive abnormalities (45).

It is worth mentioning that the hippocampus is very vulnerable to several neurotoxic insults, including fetal hypoxia and iron deficiency, both of which are phenomena reported in fetuses from diabetic mothers (46–49). One way to evaluate early postnatal hippocampal function is through the measurement of event-related potentials (ERPs) generated by visual or auditory stimuli. ERPs are divided into two categories: sensory, which are early waves, peaking within the first 100 ms after the stimulus, and cognitive, which are waves generated later due to information processing (50). Using this tool, recognition memory (visual and auditory stimuli) was evaluated in 6-month-old infants from diabetic mothers, finding robust evidence of a memory deficit (51, 52).

During embryo development, the cerebral cortex and hippocampus arise from the dorsal telencephalic neuroepithelium. As in other areas of the CNS, intrinsic and extrinsic factors coordinate correct patterning during development by promoting the self-renewal of neural stem cells (NSC). These then will specify into neurons, astrocytes, and oligodendrocytes, to develop the functional areas of the cerebral cortex and the characteristic hippocampus anatomy. Diabetes during pregnancy in mice promotes changes in the expression of proliferative and differentiative related genes during brain development. However, NTD embryos or complete litters have been used, which makes it difficult to relate changes in gene expression during fetal development and postnatal impaired cognitive functions in offspring without NTD. Moreover, contrasting data have been reported regarding the effect of hyperglycemia on embryo cell proliferation, differentiation, and survival. Thus, an increase in cell proliferation without affection on cell death, which promotes thickening and deformation of the dorsal telencephalon in embryos to diabetic mice, was reported at E11.5. However, increased BrdU incorporation was not observed (38, 39). On the other hand, there are several reports which suggest increased cell death using the same model and embryo data (53–57). Despite some discrepancies, changes in cell proliferation, migration, and differentiation may be taking place in a different and complex manner, and even depending on the embryos susceptibility. Thus, researchers need to separate non-NTD from NTD embryos in order to define both shared and different mechanisms, and, in turn, propose and establish specific prevention, treatment, and diagnosis strategies beyond NTD.

Even though the telencephalon is the most studied structure during development in health and disease, few studies have analyzed the effect of hyperglycemia in embryos with “normal” (non-NTD) development. However, the use of cortical NSC obtained from the dorsal telencephalon can be a useful tool to study the effect of hyperglycemia. Fu et al. (39), studied the effect of high glucose (30 mM) on the proliferation and differentiation of E13 cortical NSC from normal pregnant mice, and showed that high glucose in proliferative NSC promoted increased cell death and reduced cell proliferation, together with increased neuron, astrocyte, and oligodendrocyte differentiation in differentiated cells. They concluded that NSC cultured in high glucose led to cell-cycle arrest and apoptosis and influenced lineage specification, through a mechanism in which Shh, Bmps and the Notch/Delta pathway are involved (39).

The effect of high glucose on cell phenotype must be more complex, and other factors may be involved, such as micro RNAs (miRNAs, molecules with a potential use in therapeutic as well as noninvasive biomarkers), neurotransmitters and epigenetic mechanisms (chromatin modification, histone methylation, or acetylation and DNA methylation). The level of telencephalic development-related miRNAs was evaluated in serum from control and GDM women whose fetuses were not diagnosed with NTD. Interestingly, GDM led to higher levels of proliferative and neurogenic miRNAs (miR-183-5p, miR-200b-3p, miR-125-5p, and miR-1290). Moreover, gene ontology *in-silico* analysis revealed alterations in cell proliferation and neuron differentiation (5). Furthermore, an array study containing ~2,383 probes was used to analyze the *in vitro* and *in vivo* effects of high glucose in mice forebrain NSC. Results showed that high glucose deregulated 104 and 25 miRNAs *in vivo* and *in vitro*, respectively. Neurogenic miRNAs: miR-30 family, miR-125-5p, miR-124, and miR-128 were upregulated under both conditions (58). Furthermore, miR-183, considered a proliferative miRNA, was downregulated in NSC obtained in embryos from diabetic mice (59, 60). Although the authors focused on the miRNA-30 family, because it is known to be involved in schizophrenia, ASD, axon extension and guidance, and other neurodevelopmental disorders (61, 62, 63), it is clear that changes in miRNAs expression may have an extensive regulatory effect. One miRNA may regulate hundreds or thousands of RNAm, which may, in turn, affect cell proliferation, differentiation, migration, and death, through a complex network of epitrascriptomic regulation occurring in parallel.

The expression of miRNAs under hyperglycemic conditions may be regulated by epigenetic factors. To explore this circumstance under high glucose *in utero* and *in vitro*, Shyamasundar et al. (64) isolated E13.5 dorsal telencephalic tissue from diabetic and control pregnant mice litters or NSC from control mice that were maintained under normal or high-glucose conditions during 48 h (40 mM). They reported that high glucose *in vivo* and *in vitro* alters chromatin reorganization due to an increase in histone H3K9 trimethylation and global DNA methylation, and provokes a decrease in histone H3K9 acetylation. Decreased gene expression due to high glucose was expected because H3K9 can turn genes on by becoming acetylated, and can silence when methylated. However, the authors reported that high glucose in NSCs in embryos from diabetic mice promoted a significant increase in the expression of doublecortin and Pafah1b1 (Platelet-activating factor acetylhydrolase isoform 1b, subunit 1), molecules essential for neuron migration and differentiation; this effect could be explained because no change in the CpG methylation status of the gene promoter was observed (65, 55, 64). They further determined that the decreased miR-200a, miR-200b, miR-466a-3p, and miR-466d-3p miRNAs were responsible for the changes observed in Dcx and Pafah1b1 (64), suggesting the epigenetic regulation of these miRNAs. Interestingly, a lower level of mir-200b was reported in serum samples from diabetic pregnant women at the third trimester, with a negative diagnosis for NTD (5).

Neurotransmitters may also contribute to increased neurogenesis observed in the cortical neuroepithelium of embryos under hyperglycemia with non-NTD. Our group reported that histamine increases cortical and mesencephalic NSC neurogenesis through H_1_-receptor (H_1_R) activation. Both this neurotransmitter and H_1_R showed a significant increase in the cortical neuroepithelial of embryos from diabetic pregnant rats (66–68). In mammals, histamine is a neurotransmitter/neuromodulator in the adult brain, acting through G-protein coupled receptors [H_1_R, H_2_RH_3_R, and H_4_R] (69, 70). During cortical development, histamine displays high concentration as well as a high expression of H_1_R and H_2_R (71–73). On the other hand, in cortical NSC, HA promotes cell proliferation and neuron differentiation through H_1_R and H_2_R activation, respectively (66, 74–76).

In the diabetic model, the cortical neuroepithelium of embryos without NTD showed increased neurogenesis (E14) as well as histamine concentration (E14) and H_1_R expression (E12). Interestingly, the systemic administration at E12 of chlorpheniramine (H_1_R antagonist/inverse agonist) partially prevented increased dorsal telencephalic neurogenesis in embryos from diabetic rats, suggesting the participation of this receptor in the impaired neurogenesis observed in embryos from the diabetic model (68). The relevance of the above findings is also supported by evidence on the effect of antihistamine drugs on controlling glycemia under diabetic conditions (77).

## Motor behavior

**Figure 3:**
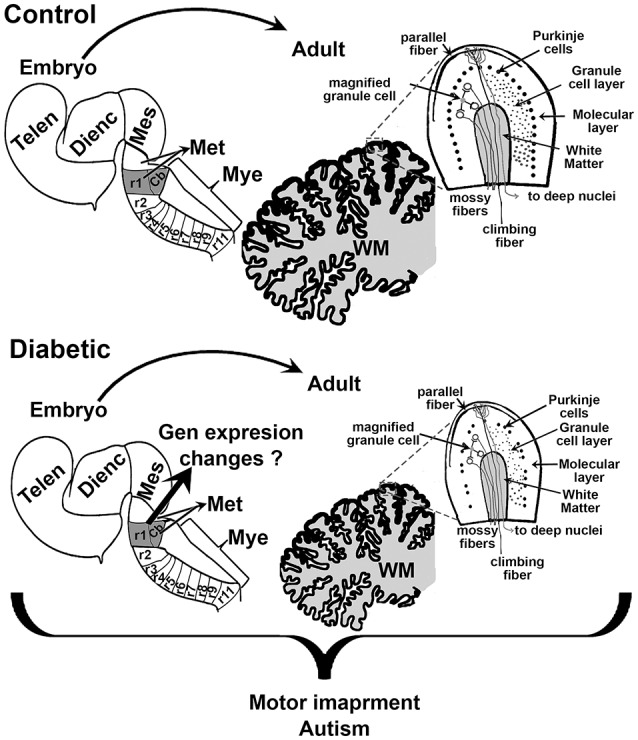
Schematic proposal of the effect of maternal diabetes on embryo and offsprings cerebellum. Sagittal views of the embryonic neural tube and adult cerebellum in control **(top)** and diabetic **(bottom)**. r1 indicates the rhombomere (rb) from which the cerebellum (Cb) rise. The scheme shows that embryos from diabetic animals present a smaller r1 and that this may reduce the size of the Cb, and the number of Purkinje and granular cells in the adult. Telen, telencephalon; Dienc, diencephalon; Mes, mesencephalon; Met, Metencephalon; Mye, Myelencephalon; WM, white matter.

Early school-age children born from mothers with GDM present motor impairment (12). As with cognition, several regions of the brain control motor activity, including the cerebral cortex and the cerebellum. As mentioned above, the dorsal telencephalon showed increased neurogenesis during fetal development. However, to our knowledge, postnatal studies on the anatomy and functional neurochemistry of the motor cortex have not been reported. In contrast, we found a few studies regarding the effect of hyperglycemia during embryo development and its effect on the cerebellum. A reduction in cerebellum size has been observed, which correlates with a decreased number of Purkinje and granular cells, and the reduced size of the molecular and granule cell layers in offspring of the streptozotocin-induced diabetic model in rats (Figure 3) (78, 79). Under these conditions, an increase in the synaptic length and dendritic spine was detected at postnatal days 30 and 70 (78), which suggests that the reduction in cerebellum size is compensated by an increase in the size of the dendritic spine. Nevertheless, it may also indicate inadequate cerebellar synaptic maturation (80).

Although postnatal cytoarchitectonic changes are reported in diabetic offspring, to our knowledge there is no evidence that could explain the postnatal changes in the cerebellum during fetal development.

## Psychiatric disorders

Autism and schizophrenia have been related to maternal diabetes. Autism spectrum disorder (ASD) is a neurodevelopmental disorder with an estimated prevalence of 1 in 68 children in 2016 (81). ASD is characterized by stereotyped behavior, severe social dysfunction, restricted attention and language impairments (82–86). Although ASD has a highly heritable factor (86), stressful environment *in utero* has also been implicated (83, 84, 86–91). In 2009, a two-fold increase in the risk of ASD in infants from GDM mothers was reported. However, authors have concluded that infections during pregnancy, maternal age, hypertension, and preeclampsia contribute to the incidence of the disease (92). This is supported by other studies that reported no relationship between GDM and risk of ASD (93, 94). However, a cohort study of 66,445 pregnancies examined the relationship of obstetric complications with ASD, finding that GDM presented a significant risk increment in the pathology of 1.2% of the children (89). A case-control study also reported that children from GDM mothers had a high incidence of ASD with expressive language deficits (84). Discrepancies among studies may be due to differences in the study design and ethnic populations.

Alterations in the cerebellum have also been related to autism (95–97). One of the most consistently abnormal findings in the postmortem brains of autistic individuals, regardless of age, sex, and cognitive ability is the significant decrease in the number of Purkinje cells in the posterolateral-neocerebellar and the adjacent archicerebellar cortices (98, 99). Both Yamano et al. and Razi et al. have shown that *in utero* hyperglycemia reduces the size of the cerebellum due to a reduction in the number of Purkinje and granular cells (78, 79). Although changes in cerebellum architecture in autism have been observed, the physiological relevance of these relationships remains unsolved, probably due to the clinical heterogeneity within the broad behavioral phenotype (100).

Genetic studies have revealed three promising ASD-implicated genes: engrailed homeobox 2 (EN2), gamma-aminobutyric acid type A receptor beta3 subunit (GABRB3), and MET proto-oncogene, receptor tyrosine kinase (MET). All have specific roles in cerebellar development (101, 102). The expression of En2 is restricted to midbrain and cerebellum. The loss of its function causes abnormal cerebellar foliation, with deficits in motor and social behavior (102). Interestingly, in embryos from diabetic mice a decrease in the expression of En2 has been reported (56, 103).

Several studies have shown a positive association between oxidative stress during fetal development and ASD, suggesting that oxidative damage is an important factor in the etiology of ASD (104, 105) and schizophrenia (106, 107). The release of reactive oxygen species (ROS) and the generation of oxidative stress under GDM is a critical aspect that may contribute.

On the other hand, schizophrenia is a chronic and severe psychiatric incapacitating disorder that affects a wide range of cognitive, emotional, and motor functions that have been associated with GDM. The symptoms include hallucinations or paranoid delusions, disorganized speech, and socialization, cognition, and memory impairment (108). The etiology of this disorder is unknown. However, epidemiological evidence suggests prenatal and perinatal complications as antecedents (109–112).

In a Swedish case-control cohort study, an increased susceptibility of developing schizophrenia in females from GDM mothers was reported, suggesting a gender susceptibility (113). A meta-analysis study reported that among the obstetric complications for schizophrenia, GDM was found to have a significant participation (114). Other studies described that there is no evidence of the association between GDM and the disease (115, 109). This inconsistency could be explained by the fact that most studies used questionnaires or scales where GDM or a history of maternal diabetes were not included.

On the other hand, it has been suggested that an increased level in plasma of pro-inflammatory cytokines such as IL-8 (interleukin-8) and tumor necrosis factor-α (TNF-α) during pregnancy could be related to offspring schizophrenia in adulthood (116). Thus, a hyperglycemic condition increases the expression of inflammatory cytokines in macrophages by decreasing H3K9me3 levels in inflammatory cytokines promoters, such as TNF-α, and chemoattractant protein-1 and IL-6 (117).

Changes in the DNA methylation pattern have been reported in term placenta and cord-blood samples from GDM. Interestingly, the analysis showed that 57 genes associated with schizophrenia or schizoaffective disorders were affected (118). Moreover, several lines of evidence have shown that alterations in the degree of DNA and histone methylation are related to ASD and schizophrenia (119–122). All these data suggest that epigenetic changes during GDM may be related to offspring schizophrenia. A “two-hit” hypothesis has been proposed for this and other diseases, where the onset of disease cannot clearly be linked to a specific genetic or environmental insult. This is also applicable to ASD. For schizophrenia, it has been hypothesized that the first hit may affect neurogenic and cell specification pathways, while the second hit may have a greater effect on functional integration (123), two processes that have been suggested to be altered in embryos and early born diabetic animals (31, 38, 39, 56).

## Conclusion

Morphological and functional alteration in the CNS and its relationship with changes in gene expression and maternal diabetes is complex. Particularly because other phenomena may be participating in the final function of a gene or group of genes, such as post-transcription, translation, or post-translational regulatory processes that define cell commitment, differentiation, migration, death, integration, and function during specific moments in development.

Although fetal programming due to maternal diabetes may be evident, and a lot of information regarding the intrinsic and extrinsic factors that participate in the development of several areas of the CNS has been generated and related to NTD, we have not developed proper experimental protocols to generate information related to how hyperglycemia affects the development of specific areas of the CNS during critical time windows. It is also clear that, depending on the timing of insult in relation to the stages of brain development, different cell populations may be selectively affected. Furthermore, it is likely that early defects may be exclusively due to hyperglycemia, while defects occurring in late development may be due to high glucose and/or hyperinsulinemia. Finally, it is important to bear in mind that the problem is complex, and that no single molecular mechanism can fully explain the effects of maternal diabetes on fetal programming during neurodevelopment. Global approaches are needed.

## Author contributions

All the authors contributed in the same way for the realization of this review. Additionally, AM-H and ND reviewed and edited the final version.

### Conflict of interest statement

The authors declare that the research was conducted in the absence of any commercial or financial relationships that could be construed as a potential conflict of interest.

## Abbreviations

GDM: gestational diabetes mellitus
NTD: neural tube defects
NPY: neuropeptide Y
POMC: pro-opiomelanocortin
AgRP: agouti-related peptide
ARC: arcuate nuclei
PVN: paraventricular nuclei
CART: cocaine and amphetamine-related transcripts
Shh: Sonic hedgehog
Nkx2.1: thyroid transcription factor 1
NSCs: neural stem cells
ASD: Autism spectrum disorder.
